# Interleukin-6 predicts inflammation-induced increase of Glucagon-like peptide-1 in humans in response to cardiac surgery with association to parameters of glucose metabolism

**DOI:** 10.1186/s12933-016-0330-8

**Published:** 2016-02-03

**Authors:** Corinna Lebherz, Florian Kahles, Katja Piotrowski, Michael Vogeser, Ann Christina Foldenauer, Kirsten Nassau, Erich Kilger, Nikolaus Marx, Klaus G. Parhofer, Michael Lehrke

**Affiliations:** Department of Internal Medicine I, University Hospital Aachen, Pauwelsstraße 30, 52074 Aachen, Germany; Department of Medical Statistics, University Hospital Aachen, Aachen, Germany; Department of Internal Medicine II, University Hospital Munich (LMU), Campus Grosshadern, Munich, Germany; Institute of Laboratory Medicine, University Hospital Munich (LMU), Campus Grosshadern, Munich, Germany; Department of Anesthesiology, Campus Grosshadern, Munich, Germany; Helmholtz Institute Munich, German Research Center for Environmental Health, Neuherberg, Munich, Germany

**Keywords:** GLP-1, IL6, Inflammation, Insulin secretion, Cardiac surgery, Inflammatory response

## Abstract

**Objective:**

Glucagon-like peptide-1 (GLP-1) is an incretin hormone, which gets secreted in response to nutritional stimuli from the gut mediating glucose-dependent insulin secretion. Interestingly, GLP-1 was recently found to be also increased in response to inflammatory stimuli in an interleukin 6 (IL-6) dependent manner in mice. The relevance of this finding to humans is unknown but has been suggested by the presence of high circulating GLP-1 levels in critically ill patients that correlated with markers of inflammation. This study was performed to elucidate, whether a direct link exists between inflammation and GLP-1 secretion in humans.

**Research design and methods:**

We enrolled 22 non-diabetic patients scheduled for cardiac surgery as a reproducible inflammatory stimulus with repeated blood sampling before and after surgery.

**Results:**

Mean total circulating GLP-1 levels significantly increased in response to surgery from 25.5 ± 15.6 pM to 51.9 ± 42.7 pM which was not found in a control population. This was preceded by an early rise of IL6, which was significantly associated with GLP-1 under inflammatory but not basal conditions. Using repeated measure ANCOVA, IL6 best predicted the observed kinetics of GLP-1, followed by blood glucose concentrations and cortisol plasma levels. Furthermore, GLP-1 plasma concentrations significantly predicted endogenous insulin production as assessed by C-peptide concentrations over time, while an inverse association was found for insulin infusion rate.

**Conclusion:**

We found GLP-1 secretion to be increased in response to inflammatory stimuli in humans, which was associated to parameters of glucose metabolism and best predicted by IL6.

**Electronic supplementary material:**

The online version of this article (doi:10.1186/s12933-016-0330-8) contains supplementary material, which is available to authorized users.

## Background

Critical illness—as found during sepsis or acute myocardial infarction—is a state of disarranged glucose metabolism featuring episodes of hyper- and hypoglycemia [1]. States of hyperglycemia are attributable to the release of stress hormones including catecholamines, cortisol and glucagon leading to increased gluconeogenesis and inflammation mediated insulin resistance [2]. States of hypoglycemia, at the other end are the consequence of an activated, hypermetabolic immune system with augmented glucose utilization, increased insulin secretion and inflammation induced suppression of gluconeogenesis [3, 4]. While hypoglycemic stimuli tend to dominate the early phase of acute inflammation, this is followed by a secondary increase of blood glucose with occurrence of insulin resistance thereafter [5]. Early inflammation dependent insulin-secretion contributes to the initial drop of blood glucose values, which was recently found to be regulated by the incretin hormone glucagon-like peptide-1 (GLP-1) [6]. GLP-1 is a gut-derived peptide, which stimulates glucose dependent insulin secretion from pancreatic beta-cells while also suppressing glucagon release from pancreatic alpha-cells [7]. GLP-1 secretion is classically induced by food ingestion and nutritional stimuli of the gut [7]. A similar induction of GLP-1 was recently found to occur in response to inflammatory stimuli including endotoxin and IL1 in mice [6]. These activate an inflammatory cascade leading to IL6 as a necessary and sufficient stimulus for inflammatory GLP-1 secretion [6]. The translatability of these findings to humans is currently unknown but is suggested by marked elevation of circulating GLP-1 levels found in critical ill patients at time of admission to the intensive care unit (ICU) [6]. Furthermore, GLP-1 was found to be associated with inflammatory markers including IL6 in ICU patients with higher GLP-1 levels being present in septic than non-septic patients [6]. Still, regulatory relevance of inflammation for GLP-1 secretion remains largely unexplored in humans. This study aims to further characterize the regulation of GLP-1 secretion by inflammatory stimuli in humans. To do so, we have performed serial assessment of GLP-1 plasma levels in 22 patients undergoing cardiac surgery with extracorporeal circulatory support as a defined and reproducible inflammatory stimulus.

## Methods

We prospectively enrolled 22 non-diabetic patients (12 male, 10 female) scheduled for cardiac surgery with cardiopulmonary bypass and requirement of extracorporeal circulation [8]. Mean age was 66 years with an BMI of 26.5 kg/m^2^. Patients were excluded from the study if they met the following criteria: pregnancy, diabetes mellitus, fasting glucose >126 mg/dl, use of antidiabetic medication or glucocorticoids. Patients were fasting since the evening of the preoperative day. Blood samples were stored on ice and directly centrifuged. Blood glucose was assessed on an hourly basis and insulin-infusion rate consequently adjusted to maintain glucose levels between 80 and 126 mg/dl. Insulin and catecholamine infusion rates were recorded at the blood collection time points. No glucose containing solutions were given during the day of the procedure, while all patients were started on a continuous infusion of glucose 10 % with a rate of 10 ml/h at the morning of the first postoperative day. No additional parenteral or enteral nutrition was supplied during the observation period. The study protocol was approved by the Ethics Committee of the Ludwig-Maximilians-University Munich, Germany. A control population of eight patients awaiting cardiac angiography was collected in the cardiology department of the university hospital Aachen with ethical approval gained by the Ethics Committee of the University Hospital Aachen. All patients gave informed written consent.

## Laboratory procedures

Blood samples were stored at −70 °C until analysis. Total GLP-1 levels were determined as described previously using chemiluminescent enzyme-linked immunosorbent assay (ELISA) [9]. Serum levels of TNFα, IL6, leptin, adiponectin and resistin were determined with a commercial ELISA (R&D, Wiesbaden, Germany). Serum concentrations of cortisol, insulin, and C-peptide were quantified using a multichannel immunoanalyzer based on electrochemiluminescence as the principle of signal generation (Roche Cobas, Elecsys 2010; Roche Diagnostics Mannheim, Germany) by the Department of Clinical Chemistry (Campus Grosshadern, University of Munich, Germany).

## Statistical analysis

**Fig. 1:**
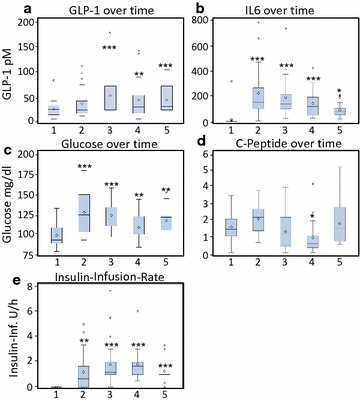
Kinetics of GLP-1 (**a**), IL6 (**b**), Glucose (**c**), C-Peptide (**d**) and Insulin-Infusion-Rate (**e**) over time with time point 1 illustrating blood drawn directly before surgery (*baseline*), time point 2: at arrival to the intensive care unit (4–6 h post initiation of surgery), time point 3: 6 h post arrival to the ICU (10–12 h post initiation of surgery) and the morning of the first (time point 4) and second (time point 5) postoperative days. *P-values* indicate the comparison to baseline (time point 1) with * <0.05; ** <0.01; *** <0.001 using a paired t-tests and Wilcoxon signed rank sum tests for the differences

For description all continuous variables are expressed as mean values ± standard deviation (SD). Correlations between baseline characteristics were assessed by the Spearman correlation coefficient which was similarly performed at later time points. Depending on the presence of normally distributed data, paired t-tests and Wilcoxon signed rank sum tests were used to compare the baseline concentration of parameters with later time points. Significant differences are presented in the boxplots (Fig. 1) and Additional file 1: Table S2. Two repeated measures ANCOVAs were performed: The first one to identify metabolic factors which show a statistical relationship to GLP-1 levels over time and the second one to evaluate the impact of GLP-1, gender, age, insulin-infusion rate and time of measurement on C-peptide. The response variables GLP-1 and C-peptide were logarithmized to meet the requirements for homoscedasticity and to acquire normally distributed data. A Kenward–Rogers adjustment was used to account for the small sample size. For the covariance a first order autoregressive structure was assumed in both analyses. For the univariable analysis of each metabolic factor a repeated measure analysis with the same assumptions was performed in advance. For the GLP-1 response, the variable selection was performed as followed: Together with the clinical factors age, gender and time of measurement, all factors that were associated with GLP-1 in the univariable analyses with a P value <0.20 were simultaneously analyzed in a multivariable model. Then, via backward selection, all factors were eliminated which impaired the model fit due to Akaike information criterion (AIC). All factors with a P value >0.20 in the univariable analyses were reconsidered by forward selection. Factors improving the AIC value were added to the model. All 2-way interactions were included in the model to explore possible effect modifiers. We assessed any effect in all tests as significant if the corresponding P value fell below the 5 % margin. All measurements of curves were fitted by locally weighted scatterplot smoothing, which uses weighted least squares to fit linear or quadratic functions of the predictors at the centers of neighborhoods (Proc Loess). Boxplots were chosen to present the fluctuations of selected factors over time. Statistical analysis was performed using SAS for Windows, Version 9.2 (SAS Institute, Cary, NC, USA), “Proc Mixed” was used for the repeated measure analysis.

## Results

All patients (n = 22) underwent open cardiac surgery with cardiopulmonary bypass. Patient characteristics are presented in Table 1. Blood samples were drawn directly before surgery (baseline), at arrival in the ICU (4–6 h time point), 6 h post arrival to the ICU (10–12 h time point) and the morning of the first and second postoperative days.

**Table 1:** Baseline characteristics of the study population (n = 22)

	Mean (±SD) or %	
Age	73	(±13)
Sex (male)	55	
Body mass index (kg/m^2^)	26.12	(±3.55)
Hypertension	55	
Smoker (yes)	32	
Operation time (min)	278	(±60.13)
Heart/lung bypass time (min)	92	(±33.93)

Mean total GLP-1 plasma concentrations did rise from 25.5 ± 15.6 pM at baseline to 36.4 ± 28.7 pM after 4–6 h to reach a maximum of 51.9 ± 42.7 pM at 10–12 h after the beginning of surgery (P < 0.0001) (Fig. 1a). No change of GLP-1 plasma concentrations were detected in a control population (n = 8) awaiting coronary angiography in the fasted state after 4 and 8 h (GLP-1 concentrations baseline: 25.4 ± 6.25 pM; after 4 h: 25.2 ± 8.37 pM; 8 h: 26.0 ± 6.57 pM). Patient characteristics of the control population are presented in Additional file 1: Table S1.

In the surgical cohort, rise of GLP-1 was preceded by an early induction of the inflammatory cytokine IL6 which peaked at the 4–6 h time point (Fig. 1b). Plasma glucose- (Fig. 1c) and C-peptide levels (Fig. 1d) were elevated at the early 4–6 h time point with a secondary decline thereafter, which was most likely attributable to exogenous insulin application (Fig. 1e) to maintain euglycemia throughout the study period.

**Fig. 2:**
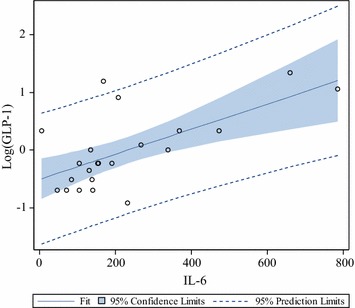
Fit-blot demonstrating the correlation of IL6 and log GLP-1 at arrival to the intensive care unit (time point 2)

Spearman coefficient analysis was used to identify parameters associated with GLP-1 at different time points. While no relevant association was found at baseline, IL6 proved to be significantly associated with GLP-1 under inflammatory conditions at time points 2–4 (Table 2 and Fig. 2). Furthermore, positive association of GLP-1 was detected with C-Peptide at a later time point and adrenalin-infusion at an early time point, while a negative association was recorded with leptin-levels at all time points after the beginning of surgery (Table 2).

**Table 2:** Spearman-Coefficient of GLP-1 with different parameters for each time point

Time-point	1	2	3	4	5
*IL6*	0.114	*0.582*	*0.490*	*0.498*	−0.058
P value	0.6150	*0.0045*	*0.0207*	*0.0182*	0.8260
*Cortisol*	0.427	0.199	−0.085	0.041	0.519
P value	0.0606	0.3743	0.7074	0.8550	0.0273
*C-Peptide*	0.360	0.179	0.406	*0.440*	−0.056
P value	0.1003	0.4252	0.0612	*0.0404*	0.8239
*Insulin*	−0.276	0.147	−0.199	−0.163	0.078
P value	0.213	0.5146	0.3744	0.4698	0.7598
*Glucose*	0.115	0.039	0.168	−0.013	0.040
P value	0.6104	0.8632	0.4558	0.9541	0.8762
*Leptin*	−0.299	*−0.605*	*−0.543*	*−0.524*	0.286
P value	0.1873	*0.0037*	*0.0109*	*0.0147*	0.2834
*Resistin*	0.218	0.134	0.187	0.166	0.424
P value	0.3418	0.5622	0.416	0.4716	0.1016
*TNFa*	0.169	0.088	−0.008	0.059	−0.252
P value	0.4632	0.7050	0.9732	0.7984	0.3640
*Adiponectin*	−0.021	0.021	−0.282	−0.239	0.121
P value	0.9335	0.9293	0.2160	0.2964	0.6559
*Adrenalin-Inf.*	−0.021	*0.820*	0.109	0.178	−0.197
P value	0.9356	*<0.0001*	0.6676	0.4793	0.4182
*Insulin-Inf.*	–	0.100	0.064	−0.002	0.498
P value	–	0.6594	0.7757	0.9931	0.0591

No insulin-infusion was present at baseline; time point 1: directly before surgery (baseline), time point 2: at arrival in the intensive care unit (4–6 h time point), time point 3: 6 h post arrival to the ICU (10–12 h time point), time point 4: morning of the first and time point 5: morning of the second postoperative day

To evaluate which parameter would best predict the rise of GLP-1 plasma concentrations over time, we performed repeated measure ANCOVA under consideration of age, sex, BMI and repetitive measurements of IL6, glucose, insulin, insulin-infusion rate, cortisol, leptin, resistin, adiponectin, TNFα and adrenalin infusion rate (Additional file 1: Table S1). Using univariate analysis we found IL6 to best predict the kinetic of GLP-1 over time (P < 0.0001), which was followed by glucose (P = 0.01) and insulin (P = 0.04) (Table 3). This was confirmed by multivariate analysis with IL6 providing the best predictor for the observed GLP-1 kinetic over time (P = 0.0009), followed by blood glucose concentrations (P = 0.03) and inversely by cortisol plasma levels (P = 0.03) (Table 4a).

**Table 3:** Univariate prediction of GLP-1 plasma levels by different factors

	F value	P value
IL6	24.59	*<0.0001*
*Glucose*	6.97	*0.0100*
*Insulin*	4.33	*0.0407*
*Adrenalin-infusion*	2.37	0.1287
*Resistin*	2.49	0.1189
*Cortisol*	1.68	0.1987
*Insulin-infusion*	1.89	0.1753
*Gender*	1.48	0.2376
*Leptin*	1.11	0.2965
*Age*	0.78	0.3885
*Adipoectin*	0.17	0.6825
*Insulin-infusion*	0.08	0.7766
*TNFα*	0.00	0.9655

**Table 4:** Multivariate prediction of (a) GLP-1 plasma and (b) C-peptide levels by different factors

	F value	P value
a. GLP-1 plasma levels		
IL6	12.25	*0.0009*
*Glucose*	5.04	*0.0293*
*Cortisol*	4.78	*0.0324*
*Age*	3.13	0.1020
*Gender*	2.01	0.1822
*Time*	1.93	0.1176
*Insulin-infusion*	1.89	0.1753
*Adrenalin-infusion*	0.54	0.4648
*TNFα*	0.24	0.6266
b. C-peptide levels		
GLP-1	7.72	*0.0071*
*Time*	5.79	*0.0004*
*Insulin-infusion*	3.91	0.0524
*Gender*	0.35	0.5613
*Age*	0.02	0.8878

To evaluate whether GLP-1 induction was secondarily associated with insulin secretion we again performed repeated measure ANCOVA using C-peptide as the dependent variable. Under further consideration of age, sex, repetitive measurement and insulin-infusion rate we found GLP-1 to significantly predict changes of C-peptide concentrations over time (P = 0.007) while a negative trend was found for insulin-infusion rate (P = 0.0524) (Table 4b).

## Discussion

In this study we used open heart surgery with extracorporeal circulatory support as a defined, reproducible stimulus to study inflammation dependent regulation of GLP-1 plasma levels in humans [8, 10, 11]. This demonstrated a time dependent increase of circulating GLP-1 levels, which recapitulates the inflammation dependent induction of GLP-1 in mice. Peak concentrations of GLP-1 were found 10–12 h post-initiation of surgery, which was relatively delayed to endotoxin dependent peak GLP-1 concentrations found 2 h after LPS application in mice [6]. This most likely reflects the differential inflammatory stimulus. In both species, GLP1 induction happened secondarily to an early rise of IL6, which is necessary and sufficient for GLP-1 secretion in mice and also proved as the best predictor for GLP-1 kinetics in humans [6, 12]. This suggests a similar, species overlapping regulative mechanism to be in place, which gets further supported by IL6 dependent increase of GLP-1 secretion from human alpha-cells [12]. Mechanistically IL6 causes STAT-3 activation leading to proconvertase 1/3—the relevant GLP-1 processing enzyme—and proglucagon expression [12]. On the contrary, GLP-1 administration has been found to reduce circulating IL6 concentrations in humans, which suggests the existence of a negative feedback loop as part of the anti-inflammatory capacities of GLP-1 [13]. Surprisingly however, administration of TNFα, as an alternative inflammatory stimulus with strong IL6 inducing potential, failed to increase GLP-1 concentrations in mice and humans [6, 14]. This illustrates the complexity of a yet incompletely understood regulative mechanism of GLP-1 secretion. We further found an early association of GLP-1 with adrenalin infusion rate, which was not present at later time points, which recapitulates the GLP-1 inducing capacity of adrenergic stimuli [15]. The mechanisms underlying the negative association of GLP-1 with leptin in this study remains to be explored and contrasts to the GLP-1 inducing capacity of leptin reported in the literature [16].

Interestingly, GLP-1 levels did predict endogenous insulin secretion as assessed by c-peptide kinetics in our study. This suggests functional relevance of inflammation induced GLP-1 secretion for insulin release in humans.

Therapies with GLP-1 receptor agonists have improved glucose control in critical ill patients. This proved effective in a small study population and lowered the need for exogenous insulin infusion with more stable glucose control and less hypoglycemic episodes [17, 18]. Given the high concentrations of endogenous GLP-1 found in critical ill patients, application of DPP-4 inhibitors might provide an alternative strategy to improve blood glucose control in this patient population.

Interestingly, GLP-1 receptor agonists and DPP-4 inhibitors were found to similarly reduce mortality in inflammatory sepsis models [19]. This might relate to their capacity to reduce the inflammatory response, which has similarly been found in rodent models and humans [19–22]. Both substances also proved capable to ameliorate the chronic inflammatory process of rodent atherosclerosis [23, 24]. Whether these immune-modulatory capacities will translate to prognostic improvement of critical ill patients treated with GLP-1 modifying therapies is currently unknown and remains to be explored.

This study has a variety of limitations. We are only able to present repeated associations of GLP-1 and other parameters in a longitudinal manner, which does not prove causality. Additional studies using IL6 modifying therapies will be required to prove the relevance of IL6 for inflammation induced GLP-1 secretion in humans. Application of DPP-4 inhibitors and/or a GLP-1 receptor antagonist will help to elucidate the relevance of inflammatory GLP-1 secretion for glucose metabolism in humans.

In conclusion, we demonstrate GLP-1 secretion to be increased in response to open heart surgery with extracorporeal circulatory support as an inflammatory stimulus in non-diabetic subjects, which was associated to factors of glucose metabolism and best explained by IL6.

## Additional file

10.1186/s12933-016-0330-8 Baseline characteristics of the control study population.** Table S2.** Kinetic of plasma parameter and infusion rate of the surgical study population over time.
