# Supplementary material for: Interleukin-6 predicts inflammation-induced increase of Glucagon-like peptide-1 in humans in response to cardiac surgery with association to parameters of glucose metabolism
Source: Cardiovasc Diabetol. 2016 Feb 3;15:21. doi: 10.1186/s12933-016-0330-8 (PMC4739342; doi:10.1186/s12933-016-0330-8)
Supplement: Supplementary file 1 — 10.1186/s12933-016-0330-8 Baseline characteristics of the control study population. Table S2. Kinetic of plasma parameter and infusion rate of the surgical study population over time. [file 12933_2016_330_MOESM1_ESM.docx]

**Table S1:**

| **Baseline characteristics of the study population (n=8)** | | | | |
| --- | --- | --- | --- | --- |
|  |  |  | Mean (± SD) or % |  |
| Age |  |  | 69,3 | (± 13) |
| Sex (male) |  |  | 87,5 |  |
| Body mass index (kg/m2) | | | 28,55 | (± 4.2) |
| Hypertension | |  | 90 |  |
| Smoker (yes) | |  | 30 |  |

**Table S2:**

Kinetic of plasma parameter and infusion rate over time (Mean ± SD)

| **Time point:** | **1** | **2** | **3** | **4** | **5** |
| --- | --- | --- | --- | --- | --- |
| **GLP-1 [pM]** | **25.5 ± 15.6** | **36.4 ± 28.7** | **51.9 ± 42.7***** | **43.5 ± 36.9**** | **43.4 ± 30.8***** |
| **Glucose [mg/dl]** | **98.7 ± 14.4** | **127 ± 28.1***** | **123.2 ± 17.0***** | **108.7 ± 14.7**** | **117.3 ± 15.8**** |
| **C-Peptide [ng/ml]** | **1.64 ± 0.85** | **2.13 ± 0.88** | **1.35 ± 0.99** | **1.00 ± 0.93*** | **1.84 ± 1.27** |
| **Insulin [uU/ml]** | **6.19 ± 6.09** | **10.3 ± 14.9** | **29.5 ± 40.8***** | **41.9 ± 45.5***** | **22.8 ± 16.6***** |
| **Insulin-Infusion-Rate [U/h]** | **0±0** | **1.18 ± 1.44**** | **1.79 ± 1.85***** | **1.81 ± 1.22***** | **1.24 ± 1.03***** |
| **Cortisol [ug/dl]** | **12.9 ± 6.7** | **17.3 ± 25.2** | **43.5 ± 26.6***** | **41.9 ± 26.6***** | **33.2 ± 26.2**** |
| **IL6 [pg/ml]** | **17.6 ± 67.8** | **225.4 ± 194.8***** | **191.6 ± 159.9***** | **144.3 ± 108.3***** | **91.8 ± 61.5**** |
| **Resistin [ng/ml]** | **13.1 ± 6.0** | **25.5 ± 9.6***** | **36.0 ± 13.3***** | **36.7 ± 16.3***** | **27.6 ± 15.3***** |
| **TNFα [pg/ml]** | **8.6 ± 3.2** | **15.4 ± 4.9***** | **13.9 ± 5.9***** | **11.8 ± 4.9***** | **12.4 ± 5.0***** |
| **Leptin [pg/ml]** | **8.18 ± 8.94** | **4.91 ± 6.89***** | **6.22 ± 8.38** | **14.83 ± 1.83**** | **9.71 ± 11.24** |
| **Adiponectin [ng/ml]** | **7.11 ± 5.60** | **4.96 ± 3.32***** | **5.08 ± 3.24***** | **4.71 ± 3.17***** | **4.75 ± 2.82**** |
| **Adrenalin-Infusion-Rate** | **0.21 ± 0.11** | **0.54 ± 0.42**** | **0.29 ± 0.29** | **0.19 ± 0.26** | **0.08 ± 0.12*** |

Indication of P-Values in comparison to time point 1 using a paired t-tests and Wilcoxon signed rank sum tests for the differences: * <0.05; **<0.01; ***<0.001; time point 1: directly before surgery (baseline), time point 2: at arrival in the intensive care unit (4 to 6 h time point), time pint 3: 6 h post arrival to the ICU (10 to 12 h time point), time point 4: morning of the first and time point 5: morning of the second postoperative day.
